# The Effects of Fungicide, Soil Fumigant, Bio-Organic Fertilizer and Their Combined Application on Chrysanthemum *Fusarium* Wilt Controlling, Soil Enzyme Activities and Microbial Properties

**DOI:** 10.3390/molecules21040526

**Published:** 2016-04-21

**Authors:** Shuang Zhao, Xi Chen, Shiping Deng, Xuena Dong, Aiping Song, Jianjun Yao, Weimin Fang, Fadi Chen

**Affiliations:** 1 College of Horticulture, Nanjing Agricultural University, Nanjing 210000, China; zhaoshuang@njau.edu.cn (S.Z.); chenxi0872@163.com (X.C.); 2011104115@njau.edu.cn (X.D.); aiping_song@njau.edu.cn (A.S.); fangwm@njau.edu.cn (W.F.); 2 Department of Plant and Soil Science, Oklahoma State University, Stillwater, OK 74078, USA; shiping.deng@okstate.edu; 3 Shanghai Honghua Horticulture Co., Ltd., Shanghai 200000, China; 2008203041@njau.edu.cn

**Keywords:** *Fusarium* wilt, disease incidence, DGGE, bacterial/fungi ratio, microbial community

## Abstract

Sustained monoculture often leads to a decline in soil quality, in particular to the build-up of pathogen populations, a problem that is conventionally addressed by the use of either fungicide and/or soil fumigation. This practice is no longer considered to be either environmentally sustainable or safe. While the application of organic fertilizer is seen as a means of combating declining soil fertility, it has also been suggested as providing some control over certain soil-borne plant pathogens. Here, a greenhouse comparison was made of the *Fusarium* wilt control efficacy of various treatments given to a soil in which chrysanthemum had been produced continuously for many years. The treatments comprised the fungicide carbendazim (MBC), the soil fumigant dazomet (DAZ), the incorporation of a *Paenibacillus polymyxa* SQR21 (*P. polymyxa* SQR21, fungal antagonist) enhanced bio-organic fertilizer (BOF), and applications of BOF combined with either MBC or DAZ. Data suggest that all the treatments evaluated show good control over *Fusarium* wilt. The MBC and DAZ treatments were effective in suppressing the disease, but led to significant decrease in urease activity and no enhancement of catalase activity in the rhizosphere soils. BOF including treatments showed significant enhancement in soil enzyme activities and microbial communities compared to the MBC and DAZ, evidenced by differences in bacterial/fungi (B/F) ratios, Shannon–Wiener indexes and urease, catalase and sucrase activities in the rhizosphere soil of chrysanthemum. Of all the treatments evaluated, DAZ/BOF application not only greatly suppressed *Fusarium* wilt and enhanced soil enzyme activities and microbial communities but also promoted the quality of chrysanthemum obviously. Our findings suggest that combined BOF with DAZ could more effectively control *Fusarium* wilt disease of chrysanthemum.

## 1. Introduction

Chrysanthemum (*Chrysanthemum morifolium*) is a commercially valuable ornamental species worldwide. Increasing urbanization and an improving standard of living is stimulating the demand for both cut flowers and pot plants in China, prompting a rise in the monoculture-based production of chrysanthemum. As for most crop species, long-term continuous monoculture-based production results in serious continuous cropping obstacles of the chrysanthemum [1,2]. In the monoculture-based production system, chrysanthemum is generally affected by the *Fusarium* wilt, caused by *Fusarium oxysporum* f. sp. *chrysanthemi* (*F. oxysporum*), which is considered to be the most important soil-borne facultative pathogen, causing economically important losses of chrysanthemum and limiting chrysanthemum production [3].

The two most effective current interventions are the fungicide carbendazim (MBC) and the soil fumigant dazomet (DAZ), but neither of these compounds is considered to be environmentally friendly and their sustained use may have negative effects on soil ecosystem [4,5]. Microbial communities and diversity in soil are seen to be critical for the maintenance of soil health and quality, while soil enzyme activities are often used as indices of microbial growth and activity in soils [6,7,8]. Soil microbial properties and enzyme activities are closely related because transformations of the important organic elements occur through microorganisms [9,10]. In addition, the repeated application of fungicides inevitably suppresses the population of beneficial soil microbes and encourages the development of genetic resistance in the pathogen. As a result, efforts are being made to elaborate biological control methods, one of which involves bio-organic fertilizers (BOFs) [11,12,13,14].

The use of BOFs has been demonstrated to reduce the incidence of soil-borne disease affecting a range of plants [12,15], although how it achieves this is unclear. Here, the effectiveness of BOFs, particularly in combination with fungicide or soil fumigation, to control chrysanthemum *Fusarium* wilt has been explored. The demonstration that this treatment regime can suppress the disease should guide the elaboration of strategies enabling the sustainable and commercially viable production of chrysanthemum, while at the same time conserving the health of the soil ecosystem.

## 2. Results

### 2.1. The Impact of Fusarium Wilt on Chrysanthemum Growth

**Figure 1:**
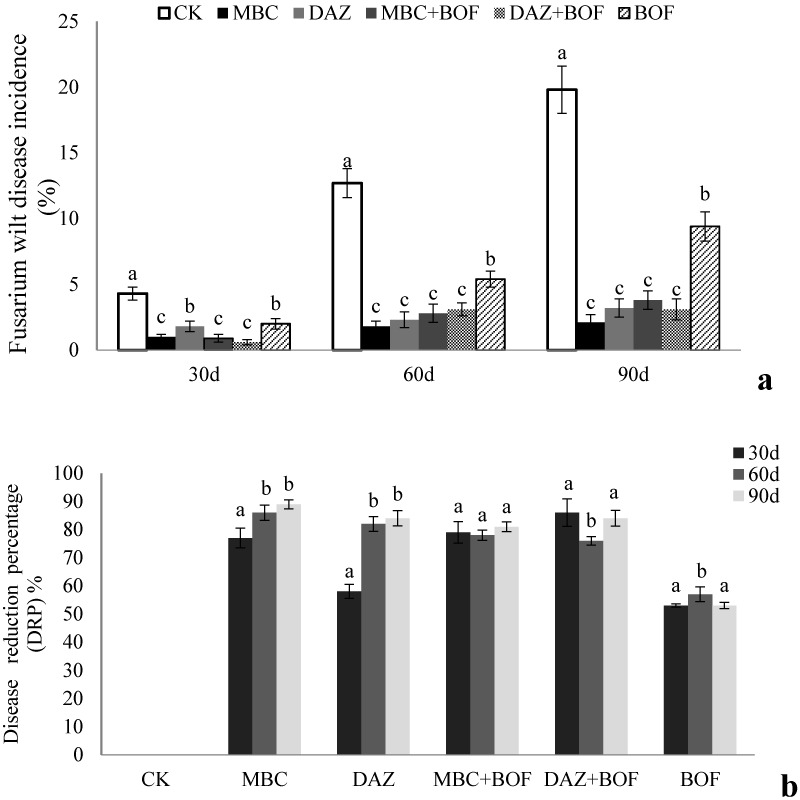
Chrysanthemum *Fusarium* wilt incidence (**a**) and disease reduction percentage (DRP) (**b**) as affected by exposure to the various treatments for between 30 to 90 days following transplantation after three croppings. The bars represent the mean and the whiskers the standard deviation. See footnote to Table 1 for the treatment codes. Letters above the bars indicate a significant difference according to Duncan’s multiple range test at *p* < 0.05 level.

Each of the treatments significantly reduced the incidence of *Fusarium* wilt (Figure 1a) and increased the disease reduction percentage (DRP) (Figure 1b). After the 90 days growth of the third cropping period (February 2012), the DI was 19.8% in the CK plots, 9.4% in the BOF plots and <4% in the other plots. The DRP was 0% in the CK plots, 89% in the MBC plots, 84% in the DAZ plots, 81% in the MBC + BOF plots, 84% in the DAZ + BOF plots, and 53% in the BOF plots (Figure 1b). Shoot height were greatest for plants exposed to the DAZ + BOF treatment, followed by MBC + BOF and BOF, and lowest in the CK treatment (Table 1). It was significantly greater in the DAZ + BOF treatment than in either the DAZ or the BOF ones. MBC was not significantly different from that recorded in the DAZ treatment. A similar response was recorded for all the shoot, leaf and flower traits, excluding the number of ray floret.

### 2.2 Enzymatic Activity

**Figure 2:**
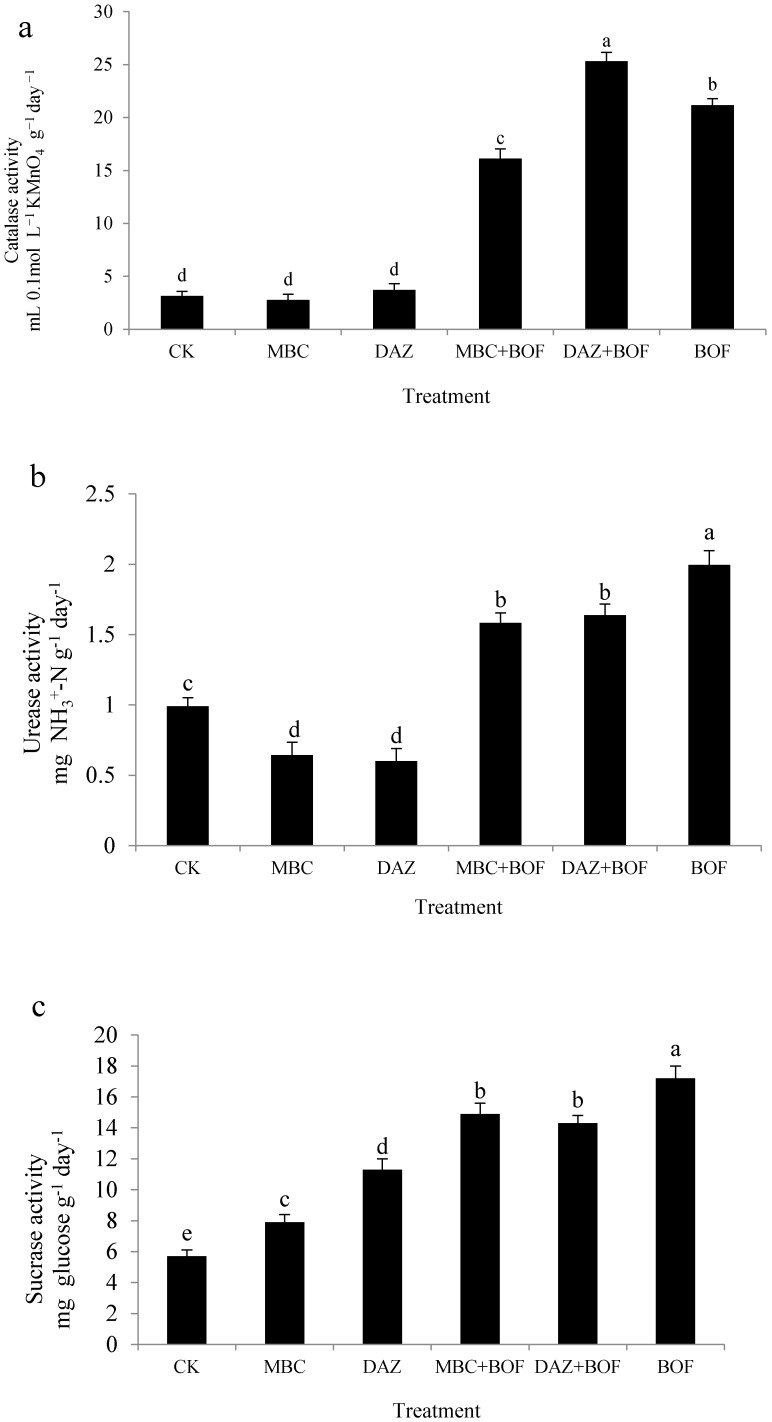
Enzyme activity: (**a**) Catalase; (**b**) Urease; and (**c**) Sucrose in soils sampled from plants experiencing the range of treatments. The bars represent the mean and the whiskers the standard deviation. See footnote to Table 1 for the treatment codes. Letters above the bars indicate a significant difference according to Duncan’s multiple range test at *p* < 0.05 level.

Soil catalase activity (Figure 2a) was significantly altered in the BOF including treatments when compared to the CK treatment, while those of both urease (Figure 2b) and sucrase (Figure 2c) were significantly affected by each of the treatments. Catalase activity was highest in the DAZ + BOF treatment (7.2 fold that of the CK soil), followed by the BOF treatment (6.1 fold) and the MBC + BOF treatment (4.6 fold). The behavior of urease was quite different: the MBC and DAZ treated soils displayed a reduced activity (respectively, 65% and 61% of the CK soil level), while the activity level was doubled in the BOF treated soil. The urease activity was also enhanced in the MBC + BOF and DAZ + BOF treated soils. Sucrase activity was highest in the BOF treated soil and lowest in the MBC treated soil if excluding CK. When BOF was combined with either MBC or DAZ, sucrase activity was suppressed compared to the level displayed in the BOF treatment.

### 2.3. Diversity of the Soil Microbiota

**Figure 3:**
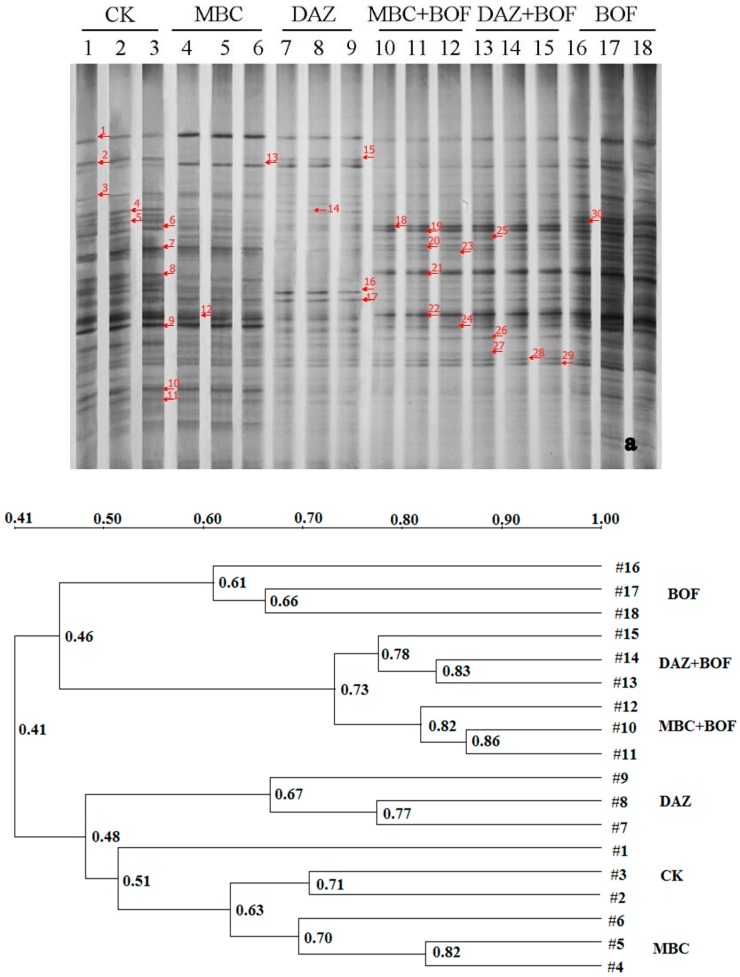

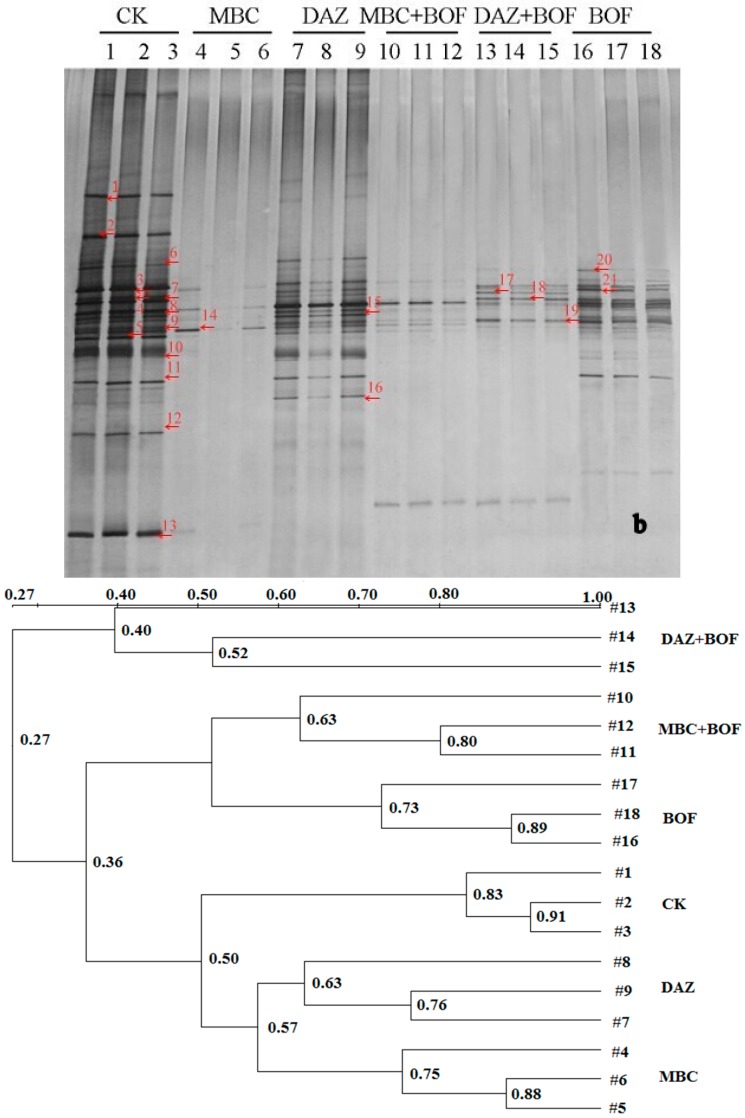
DGGE profiling of soil microbiota: (**a**) the bacterial community; and (**b**) the fungal community. Numbered fragments were excised, re-amplified and sequenced. See footnote to Table 1 for the treatment codes.

Based on the real-time PCR outputs, all of the treatments led to an increased presence of bacteria but a decreased presence of fungi, resulting in a marked increase in the ratio of bacteria to fungi of up to 30.5 (Table 2). The DAZ + BOF treatment was the most conducive for bacterial growth, followed by MBC+BOF and BOF, while the DAZ treatment was the most suppressive for fungi. The bacterial and fungal DGGE profiles fell into six and seven clusters, respectively (Figure 3). The bacterial (Figure 3a) communities of BOF were clustered together at the level of the similarity coefficient of 61%, while the fungal communities (Figure 3b) in those soils in which DAZ + BOF formed part of the treatment were clustered together with the similarity coefficient of 52%, except #13 (40%), this may have been due to an acceptable soil sampling variation. The number of fragments detected ranged from 24 to 35 (bacteria) and 5 to 24 (fungi) (Table 3). *H′* values suggested rather limited variation in the bacterial community (a range in *H′* from 3.12 to 3.33), while the range relating to the fungal component was 1.44~3.00. The highest bacterial *H′* value was recovered in the soils treated with BOF, whereas the highest fungal one was present in the non-treated control soil. The *J* metric was in general higher for the bacterial than for the fungal component. All of the treatments showed no significant differences on *J* related to the bacterial evenness, but there was no such consistency for the fungal community (Table 3).

According to the NCBI taxonomy database, 30 bacterial DGGE fragments were excised for sequencing, only one shared complete homology with a known sequence, present in a *Flavobacterium* sp. (Table 4). The remaining 29 were 94%–99% homologous with known sequences; 14 involved sequences present in uncultured *bacteria*, and the other 15 to sequences present in *Alpha proteobacterium*, *Rhizobium* sp., *Variovorax* sp., *Anoxybacillus*, *Microbacterium* sp., *Sphingobium yanoikuyae*, *Flavobacterium* sp., *Dechlorospirillum* sp., *Ochrobactrum* sp. and *Clostridium neonatale*. Of the 21 sequenced fungal DGGE fragments, one was fully homologous with an *Ophiobolus herpotrichus* sequence (Table 5), while 18 of the remaining 20 shared 96%–99% homology with sequences present in *Aspergillus terreus*, *Leptosphaeria maculans*, *Fusarium* sp., *O. herpotrichus*, *Phoma* sp. and *Phoma macrostoma*; the sequences of the other two shared homology with sequences present in uncultured soil fungi.

## 3. Discussion

The control of *Fusarium* wilt is a priority for the sustainable production of chrysanthemum. Utilization of compost to reduce the addition of chemical fertilizers and fungicides in plant protection is a promising strategy for both the present and future. The inclusion of BOF among the soil treatments evaluated was based on the expectation that it could not just shift the composition of the soil microbiota in a way which suppresses the *Fusarium* pathogen, but also could enhance soil quality and thereby plant productivity. In the event, the treatment that combined BOF with a moderate application of DAZ was the most effective in controlling the disease, improving the growth of the chrysanthemum plants, and maintaining the diversity of the soil microbiota.

### 3.1. The Effects of the Soil Treatments on Fusarium Wilt Incidence

The combined BOF/MBC and BOF/DAZ treatments’ superiority are fully in line with similar reports showing that combining chemicals with a biocontrol agent can effectively suppress soil pathogens [4,15]. One suggestion is that the provision of BOF supports the growth of pathogen antagonists [16], however the treatment involving BOF on its own did not markedly reduce the incidence of *Fusarium* wilt. This failure may have been due to the presence of a high pathogen load in the soil, given that chrysanthemum had been continuously cropped at the site for many years, so that any beneficial effect of the BOF on the growth of the pathogen antagonists may have been insufficient to overcome the pathogen pressure [16,17].

The applications of either MBC or DAZ on their own lowered the disease incidence and heighten the disease reduction percentage, but both interventions were significantly inhibitory over the urease enzyme activity of the soil microbiota. Because many soil enzymes are highly responsive to soil disturbance, their activity has been used as an index of environmental stability and soil quality. Given that the urease activity tended to fall in response to either MBC or DAZ, it is important that temporal changes in enzyme activity should been considered when assessing the sustainability of a disease control intervention.

In contrast, the application of BOF enhanced catalase, sucrase and urease activity. Previous studies have shown that both fungicide application and soil fumigation tend to reduce soil enzyme activity, presumably because of the treatments’ downward pressure on the viability of soil microbiota [7,18,19,20].

### 3.2. The Effect of the Various Soil Treatments on Soil Microbiota Composition

The DGGE-based diversity profiling indicated that BOF and various combinations of fungicides significantly affected the structure of the soil microbiota. The reduced number of fungal DGGE fragments detected in the BOF/DAZ and BOF/MBC treated soil showed that species richness was compromised, but not as severely as in soil treated with MBC. The same conclusions could be drawn from the behavior of the other diversity metrics *H′* and *J*, supporting the observation reported by [21] that BOF application encourages the growth of *Paenibacillus* spp., but decreases the level of fungi diversity. The likelihood is that the organic nutrients supplied by the manure favored the growth of particular soil microbiota species [21,22].

The application of BOF induced significant changes to the composition of the soil microbiota. Most of the 30 sequenced bands of 16S rDNA in this study were related to soil bacterial such as *S. yanoikuyae*, *Mycobacterium gilvum*, *Variovorax* sp. and *C. neonatale*, along with species in the genera *Dechlorospirillum*, *Ochrobactrum* and *Flavobacterium*. According to the literature, both *Microbacterium* sp. and *Sphingobium* are effective degraders of certain herbicides [23,24]. *S. yanoikuyae* is an efficient carbazole-degrading organism which has been exploited in the context of a number of biotechnology applications [25,26]. A strain of *Variovorax* sp. has been shown to be capable of using certain fungicides as a source of energy and nutrients [27]. On the basis of the fungal-specific 18 S rRNA assay, most of the 21 sequenced bands of BOF including treatments were related to soil fungi such as *L. maculans*, *O. herpotrichus*, *Aspergillus ochraceus*, *P. macrostoma* and various *Fusarium* sp. and other *Phoma* sp. These fungi are common in soil and many of them have been associated with phytopathogenesis [28,29].

The combined BOF/DAZ treatment resulted in a large increase to the B/F ratio, which reached 1.6 fold that resulting from the DAZ treatment and 1.5 fold that resulting from the BOF/MBC treatment (Table 2); possible explanations for this are that the combined BOF/DAZ treatment changed the composition of the soil microbiota [13,16,30]. BOF has been reported to have significant effects on population of pathogenetic fungi in cucumber [31,32], banana [14,33], cotton [13], and watermelon [16]. In watermelon, for example, the provision of BOF during the seedling stage can significantly attenuate the density of soil pathogens, an observation that suggests the possibility that antibiotics are being produced by antagonistic microorganism [28,29]. In cotton, BOF effectively controls *Verticillium* wilt in cotton by suppressing the size of the *V. dahlia* population [30]. In addition, fungicides act by inhibiting fungal growth [4,34], with consequential effects on the B/F ratio.

### 3.3. The Effect of the Various Treatments on Chrysanthemum Productivity

The major finding from the present experiments has been that combining BOF with a modest dose of DAZ or MBC encouraged plant growth more strongly than was achievable via the use of MBC or DAZ on its own. The combined treatment significantly enhanced both vegetative and reproductive growth, as has been similarly shown in watermelon plants challenged by *Fusarium oxysporum* [16]. The effect is likely related to a combination of the nutrients present in the BOF, the activity of pathogen antagonists that thrive in the BOF, and the production of antibiotics produced by members of the *Paenibacillus* genus [21,28,29]. Organic amendments are routinely used to improve soil structure and plant nutrition; the BOF used provided a ready source of nutrients to support both plant growth and the establishment of *Paenibacillus* spp. populations. Certain peptides produced by *P. polymyxa* (SQR-21) have demonstrated antibiosis against *F. oxysporum* and other pathogenic fungi [28].

## 4. Materials and Methods

### 4.1. Site Description and Plant Material

The experimental site was located in Shanghai, China. Prior to the initiation of the trial, chrysanthemum production had been carried out uninterrupted over nine years without any chemical intervention against soil-borne disease, and the incidence of *Fusarium* wilt was high. From April 2011 to February 2012, three consecutive croppings (April to June 2011, August to October 2011, and December 2011 to February 2012) of chrysanthemum were grown. The soil (a sandy loam) had a pH of 7.2, and an average content of 11.3 g organic matter·kg^−1^, 1.32 g·N·kg^−1^, 0.11 g available P·kg^−1^, and 8.18 g available K·kg^−1^. Seedlings of the chrysanthemum cultivar ‘Jinba’ (provided by Honghua Horticulture Co. Ltd., Shanghai, China) were established in a greenhouse in a perlite medium at spacing of 20 cm for three weeks under a 16 h photoperiod and a relative humidity of 70%, with the daytime temperature held at 28 °C and the nighttime temperature at 22 °C.

### 4.2. BOF and Fungicide and Soil Fumigant

Prior to its incorporation in the soil, the antagonistic microbe *Paenibacillus* *polymyxa* SQR21 (a bacterial strain known to be a highly efficient antagonist against *F. oxysporum*) was used to prepare bioorganic fertilizer (BOF) [17]. The antagonist was grown in beef extract and peptone liquid culture on a shaker at 170 rpm at 30 °C for 2–3 days. This culture was then used directly to prepare the BOF product as described below. Organic fertilizer (OF), used for the BOF product, was composed of amino acid fertilizer and pig manure compost (1:1, *w*/*w*). Amino acid fertilizer was made from oil rapeseed cakes that were enzymatically hydrolyzed by aerobic microbial fermentation at <50 °C for 7 days. This amino acid fertilizer was containing 44.2% organic matter and 12.9% of amino acids, small molecular peptides and oligo peptides. The nutrient contents were 4.4% nitrogen (N), 5.27% available P, and 0.78% available K. Pig manure compost was made by Tian-niang Ltd. (Yixing, China) in Suzhou by composting pig manure at a temperature range of 30–70 °C for 25 days. This compost was contained of 30.4% organic matter, 2.0% N, 1.6% available P, and 1.2% available K. The organic fertilizer, which was enriched with *P. polymyxa* SQR21, was named bio-organic fertilizer (BOF).

The BOF used in this experiment was obtained by aerobically fermenting OF with the SQR21 for 6 days at <45 °C. The mature BOF product contained approximately 5 × 10^9^ CFU·g^−1^ dry weight (DW) of SQR21, the nutrient contents were 59% organic matter, 5.1% N, 5.8% available P, and 1.23% available K. The BOF product was stored at less than 25 °C prior to use in experiments [17].

The fungicide carbendazim (methyl 2-benzimidazole carbamate, MBC, pure ≥ 99.5%) 50% WP and the soil fumigant dazomet (3,5-dimethyl-1,3,5-thiadiazinane-2-thione, DAZ, pure ≥ 95.0%) were purchased from Yuelian Chemical Industry Co. Ltd. (Shanghai, China) and Shizhuang Chemical Industry Co. Ltd. (Nantong, China).

### 4.3. Soil Treatment and Sampling

The experiment was a randomized complete block design with three replicates and six treatments, which resulted in a total of 18 plots. Before planting, the soil was plowed to a 25 cm depth. Each plot was 80 cm × 100 cm, holding 80 plants. The six treatments were: (1) an untreated control (CK); (2) 25 g MBC per m^2^ applied by watering the soil; (3) 30 g DAZ per m^2^ soil, applied by mixing the DAZ microgranules into the top 15 cm of the soil, after which the soil was covered by a plastic membrane for 20 days and left exposed for a further 7 days prior to planting; (4) 25 g MBC per m^2^ + 2.5 kg BOF per m^2^, 7 days after treated soil with 25 g MBC per m^2^, soil was further amended with the BOF at a rate of 2.5 kg·m^−2^; (5) 30 g DAZ + 2.5 kg BOF per m^2^, after treated soil with 30 g DAZ per m^−2^ (DAZ application method as described in treatment 3), soil was further amended with the BOF at a rate of 2.5 kg·m^−2^; and (6) 2.5 kg BOF per m^2^. Finally, chrysanthemums from the third croppings with various treatments were obtained in February 2012. Ten plants were sampled randomly from each replicate 90 days after transplanting (February 2012). The adhering soil was gently shaken off each sampled plant, passed through a 2 mm sieve and stored at 4 °C prior to analysis.

#### 4.3.1. The Measurement of Growth and Disease Incidence

Plant growth was monitored with respect to shoot height and diameter, shoot fresh and dry weight, leaf width and length, flower diameter and ray floret number. Leaf chlorophyll content (SPAD value) was quantified using a SPAD-502 Plus chlorophyll meter (Top Instrument Co., Hangzhou, China). Infection by *F. oxporum* was checked on a daily basis. A disease incidence (DI) score for each plot was assigned as the ratio of infected plants present at 90 days (January 2012), and the disease reduction percentage (DRP) was given by the expression (1 − D_T_/D_C_) × 100, where D_C_ and D_T_ were the DI values in the CK and treatment plots, respectively (Ling *et al.*, 2012).

#### 4.3.2. Soil DNA Extraction and PCR Amplification

DNA was extracted from the soil samples in triplicate, using an Ultra Clean™ Soil kit (MOBIO Laboratories, Carlsbad, CA, USA). The DNAs from the replicates were pooled and subjected to a spectrophotometric evaluation of quality and concentration using a NanoVue device (GE Life Sciences, Piscataway, NJ, USA). To detect the fungal content of the soil DNA, the ITS region of the fungal 18S rRNA sequence was amplified from this DNA using the primer pair ITS1-F/ITS2 [17,35]. Each 50 µL PCR contained 5 µL 10× PCR buffer (Mg^2+^ free), 3 µL 25 mM MgCl_2_, 4 µL 2.5 mM dNTP, 1 U Taq DNA polymerase (Takara, Dalian, China), 2 µL of each primer (10 nM) and 4 µL DNA template (25 ng/µL). The PCR regime comprised a 95 °C/5 min denaturation, followed by 35 cycles of 95 °C/50 s, 57 °C/60 s, 72 °C/60 s, and was completed by final extension of 72 °C/10 min. Similar reactions were used to amplify a fragment of the bacterial 16S rRNA sequence, using the primer pair Eub338/Eub518 [36]. A GC clamp (5′-CGCCCGCCGCGCGCGG CGGGCGGGG CGGGGGACGGGGGG) was added to the 5′ end of both ITS1-F and Eub338 to stabilize the melting behavior of the amplicon. The PCR products were purified using an Axyprep™ DNA gel extraction kit (Axygen Biotechnology Limited, Hangzhou, China) prior to their electrophoretic separation.

#### 4.3.3. Denaturing Gradient Gel Electrophoresis (DGGE) Profiling

The 18S and 16S rRNA amplicons were subjected to DGGE using the D-Code DGGE system (Bio-Rad, CA, USA). A 20 µL aliquot of the PCR product (containing ~400 ng DNA) was loaded onto each lane of a DGGE gel; for the fungal amplicons, the polyacrylamide concentration was 8% (37.5:1 acrylamide/bisacrylamide) and the denaturing gradient was 25%~45%; for the bacterial amplicons, the polyacrylamide concentration was 7% (37.5:1 acrylamide/bisacrylamide) and the denaturing gradient was 30%–70%. The electrolyte was 40 mM Tris acetate, 1 mM EDTA, pH 8.0. The temperature was held at 60 °C and the voltage at 80 C for 16 h. The amplicons were visualized by silver staining, following [17]. Prominent fragments were excised and sequenced.

#### 4.3.4. Real-Time PCR

Real-time PCR was conducted to estimate the abundance of bacterial and fungal species. The reactions were based on 2× TaKaRa SYBR^®^ Premix Taq™ (Bio technology Dalian Co. Ltd., Dalian, China) using a Mastercycler ep realplex 2S device (Eppendorf, Germany). The primer pairs were Eub338/Eub518 (bacteria) and ITS1-F/ITS2 (fungi). Each 25 μL reaction contained 10 µL 2× SYBR^®^ Premix ExTaq™, 0.5 µL of each primer, 0.5 μL 50× ROX Reference Dye II and 2 µL DNA. For the bacterial assay, the PCR comprised an initial denaturation of 95 °C/10 min, followed by 40 cycles of 95 °C/10 s, 54 °C/10 s, 72 °C/30 s, while for the fungal amplicons, the initial denaturation step was 95 °C/5 min and the cycling regime was 45 cycles of 95 °C/15 s, 53 °C/30 s, 72 °C/45 s. Standard curves were generated using a 10-fold serial dilution (from 10^9^ to 10^4^ copies) of a plasmid containing a full length copy of *Saccharomyces cerevisiae* 18S rRNA or *Escherichia coli* 16S rRNA [3,14]. Plasmid was extracted by Axyprep™ Plasmid Miniprep Kit (Axygen, Hangzhou, China). Each biological sample was subjected to three technical replicates.

#### 4.3.5. Enzymatic Activity

Catalase activity was determined using a titrimetric method [37]. The residual H_2_O_2_ was determined by titrated with KMnO_4_ in the presence of H_2_SO_4_ after 30 min of reaction. Catalase activity was expressed as mL 0.1 mol·L^−1^ KMnO_4_ consumed g^−1^·soil day^−1^. Urease activities were determined according to a method described by Liu *et al.* [38] and Fang *et al.* [39]. Urease activity was expressed as mg NH_3_–N released g^−1^·soil day^−1^. Sucrase activity was determined by a method described by Guan [37] with minor modifications. Briefly, a 5 g soil sample was treated by the addition 1 mL of toluene, followed by 15 mL sucrose and 5 mL phosphate buffer [mix 94.6 mL of Solution A (dissolve 13.61 g of KH_2_PO_4_ in water and dilute to 1 L) and 3.6 mL of Solution B (dissolve 35.81 g of NaH_2_PO_4_ in water and dilute to 1 L, pH 5.5)]. After incubation at 37 °C for 24 h, the suspension was filtered through Whatman 1001-090 filter paper (Whatman International, Maidstone, UK) and 0.5 mL of filtrate was treated with 1.5 mL salicylic acid and held for 5 min at 100 °C. Once the solution had cooled, sufficient deionized water was added to make the volume up to 25 mL. The intensity of the pink color that developed after 60 min was measured spectrophotometrically at 508 nm, using a UV2300 device (Shanghai Zhongchen instrument Co., Shanghai, China). The quantity of reducing sugar released by sucrase activity was determined by reference to a calibration curve and expressed as mg glucose g^−1^·soil day^−1^.

### 4.4. Statistical Analyses

One- and two-way analyses of variance (ANOVA) were conducted to compare enzymatic activities and microbial abundance in the soil samples. The Duncan multiple range test was used to assign significance to differences (*p* < 0.05) between treatment means. All statistical analyses were carried out using SPSS v20.0 software (SPSS, Chicago, IL, USA). Silver stained DGGE bands were analyzed with Quantity One computer soft-ware (version 4.6.3, Bio-Rad, Hercules, CA, USA). The relative intensity of individual DGGE fragments was expressed as the ratio between the intensity of that band and the total intensity of all bands in that lane. A Shannon index (*H′*) was calculated from the DGGE profiles following [40]. The Shannon–Weaver equitability index (*J*) was derived from the expression *H′*/(Ln *ni*), where *n_i_* was number of species detected in a given soil sample.

## 5. Conclusions

Treatment with either fungicide or soil fumigation, and particularly in combination with the provision of BOF, provided good control over *Fusarium* wilt. BOF including treatments show good performance in producing the best quality of chrysanthemum, but the BOF/DAZ combination was the most efficacious in terms of increasing the B/F ratio and enriching the diversity of the soil microbiota. These results will guide the improvement of strategies against *Fusarium* wilt of chrysanthemum.

## Figures and Tables

**Table 1:** The effect of the various soil treatments on the growth of chrysanthemum *.

Treatment	Shoot				Leaf			Flower	
	Height (cm)	Diameter (cm)	Fresh wt (g/plant)	Dry wt (g/plant)	Width (cm)	Length (cm)	SPAD Value (%)	Diameter (cm)	Ray Floret Number (No.)
CK	56.45 ± 4.01 ^d^	3.35 ± 0.32 ^d^	49.31 ± 4.75 ^d^	8.95 ± 0.89 ^d^	3.24 ± 0.31 ^d^	6.48 ± 0.55 ^d^	13.67 ± 1.73 ^d^	8.25 ± 0.46 ^d^	231.83 ± 17.34 ^f^
MBC	62.63 ± 3.32 ^c^	4.39 ± 0.41 ^c^	56.61 ± 4.57 ^c^	9.97 ± 0.73 ^c^	4.01 ± 0.24 ^c^	7.46 ± 0.46 ^c^	14.92 ± 1.66 ^c^	9.32 ± 0.52 ^c^	249.20 ± 18.56 ^e^
DAZ	63.01 ± 3.49 ^c^	4.91 ± 0.34 ^c^	57.05 ± 5.52 ^c^	10.16 ± 0.87 ^c^	4.28 ± 0.27 ^c^	7.51 ± 0.38 ^c^	14.53 ± 1.62 ^c^	9.39 ± 0.43 ^c^	259.55 ± 14.39 ^d^
BOF	69.83 ± 4.18 ^b^	5.92 ± 0.37 ^b^	68.79 ± 4.28 ^b^	14.23 ± 0.83 ^b^	5.43 ± 0.29 ^b^	8.32 ± 0.42 ^b^	17.35 ± 1.71 ^b^	10.50 ± 0.51 ^b^	261.89 ± 17.77 ^c^
MBC + BOF	69.98 ± 4.41 ^b^	5.99 ± 0.35 ^b^	69.08 ± 4.98 ^b^	14.76 ± 0.64 ^b^	5.27 ± 0.31 ^b^	8.45 ± 0.34 ^b^	17.07 ± 1.63 ^b^	10.43 ± 0.49 ^b^	269.34 ± 21.97 ^b^
DAZ + BOF	72.01 ± 3.92 ^a^	6.96 ± 0.33 ^a^	72.92 ± 4.82 ^a^	16.17 ± 0.91 ^a^	6.34 ± 0.25 ^a^	9.46 ± 0.36 ^a^	18.39 ± 1.92 ^a^	11.49 ± 0.40 ^a^	271.49 ± 19.72 ^a^

* Data given in the form mean ± standard deviation. Treatment codes: CK: No treatment, MBC: Fungicide carbendazim treatment, DAZ: fumigation dazomet treatment, MBC + BOF: Fungicide carbendazim and BOF treatment, DAZ + BOF: fumigation dazomet and BOF treatment, BOF: Bio-organic fertilizer treatment. SPAD value means the leaf chlorophyll content. Different letters indicate significant differences among soil treatments according to Duncan’s multiple range test at *p* < 0.05 level.

**Table 2:** Quantification of soil bacteria and fungi populations based on real-time PCR. Values given in the form mean ± SE (*n* = 3) *.

Treatment	Bacteria (10^4^ cfu·g^−1^ soil)	Fungi (10^4^ cfu·g^−1^ soil)	Bacteria/Fungi Ratio
CK	89.6 ± 0.59 ^c^	8.66 ± 0.39 ^a^	10.3 ± 0.9 ^d^
MBC	92.8 ± 0.48 ^c^	7.34 ± 0.37 ^bc^	13.7 ± 1.0 ^c^
DAZ	91.7 ± 0.68 ^c^	4.94 ± 0.22 ^d^	18.6 ± 1.5 ^b^
MBC + BOF	145.0 ± 0.72 ^b^	6.78 ± 0.39 ^b^	19.8 ± 1.2 ^b^
DAZ + BOF	179.0 ± 0.73 ^a^	5.86 ± 0.38 ^c^	30.5 ± 1.1 ^a^
BOF	140.0 ± 0.82 ^b^	6.47 ± 0.42 ^bc^	21.6 ± 1.2 ^b^

* See footnote to Table 1 for the treatment codes. Different letters indicate significant differences among soil treatments according to Duncan’s multiple range test at *p* < 0.05 level.

**Table 3:** Shannon diversity (*H'*) and equitability (*J*) indices of the soil microbiota in response to the various soil treatments. The indices and number of fragments (*n*) were determined from DGGE profiles. Data given in the form mean ± SE (*n* = 3) *.

Treatments	Bacteria			Fungi		
	*n*	*H′*	*J*	*n*	*H′*	*J*
CK	25	3.10 ± 0.30 ^a^	0.96 ± 0.02 ^a^	24	3.00 ± 0.01 ^a^	0.66 ± 0.07 ^d^
MBC	32	3.15 ± 0.33 ^a^	0.98 ± 0.09 ^a^	5	1.24 ± 0.10 ^f^	0.77 ± 0.06 ^c^
DAZ	24	3.12 ± 0.23 ^a^	0.98 ± 0.04 ^a^	16	1.65 ± 0.12 ^b^	0.81 ± 0.02 ^b^
MBC+BOF	25	3.14 ± 0.37 ^a^	0.97 ± 0.03 ^a^	7	1.81 ± 0.18 ^e^	0.89 ± 0.00 ^b^
DAZ+BOF	26	3.19 ± 0.11 ^a^	0.97 ± 0.02 ^a^	8	1.99 ± 0.20 ^d^	0.96 ± 0.01 ^a^
BOF	35	3.23 ± 0.22 ^a^	0.96 ± 0.11 ^a^	14	2.57 ± 0.11 ^c^	0.98 ± 0.04 ^a^

* See footnote to Table 1 for the treatment codes. Different letters indicate significant differences among soil treatments according to Duncan’s multiple range test at *p* < 0.05 level.

**Table 4:** Phylogeny of sequences present in the 16S rRNA amplicons (bacteria).

DGGE Band	Closest Relatives Microorganisms (Phylogenic Affiliations)	Similarity (%)	Genebank Accession No.
1	Uncultured *Xanthomonadaceae* bacterium	96	FJ53688611
2	Uncultured *bacterium*	97	EU362858.1
3	Uncultured *Ohtaekwangia* sp.	97	JX493344.1
*4*	Uncultured *Bacillus* sp.	99	HQ1791481
5	*Alpha proteobacterium*	96	JQ608334.2
6	Uncultured *Bradyrhizobium* sp.	95	HE654679.1
7	Uncultured *Xanthomonadaceae* bacterium	97	FJ88933811
8	Agricultural soil *bacterium*	98	HQ132702.1
9	*Uncultured beta proteobacterium*	98	AJ318162.1
10	*Uncultured* *methylovirgula* sp.	99	KC297188.1
11	*Alpha proteobacterium*	97	AB470422.1
12	*Rhizobium* sp.	95	HG423545.1
13	*Variovorax* sp.	97	HM484318.1
14	*Microbacterium* sp.	98	AJ318162.1
15	*Sphingomonas* sp.	98	JQ608334.2
16	Uncultured *bacterium*	97	FJ796671.1
17	*Anoxybacillus flavithermus*	98	KF279366.1
18	*Sphingobium yanoikuyae*	98	GQ214010.1
19	Uncultured *Chloroflexi bacterium*	94	HM164420.1
20	*Flavobacterium* sp.	100	KF891387.1
21	*Uncultured bacterium*	96	JQ890611.1
22	*Dechlorospirillum*	98	GU167977.1
23	Uncultured *Flexibacter* sp.	96	GU201555.1
24	*Ochrobactrum* sp.	97	HQ659714.1
25	Agricultural soil *bacterium*	95	KC193578.1
26	*Uncultured bacterium*	99	EU419388.1
27	*Mycobacterium* sp.	97	DQ658940.1
28	Uncultured *Methylocystis* sp.	97	GU227561.1
29	*Mycobacterium gilvum*	97	AJ699170.3
30	*Clostridium neonatale*	96	GU227558.1

**Table 5:** Phylogeny of sequences present in the 18S rRNA amplicons (fungi).

DGGE Band	Closest Relatives Microorganisms (Phylogenic Affiliations)	Similarity (%)	Genebank Accession No.
1	*Aspergillus terreus*	98	JN639854.1
2	*Leptosphaeria maculans*	98	NW003533867.1
3	*Fusarium* sp.	97	EU381149.1
4	*Ophiobolus herpotrichus*	100	U43453.1
5	*Psilocybe silvatica*	97	DQ851583.1
6	*Alnicola* sp.	98	JN939094.1
7	Uncultured *Clitopilus*	96	GQ995701.1
8	*Mythicomyces corneipes*	98	DQ092917.1
9	*Arthrobotrys oligospora*	98	JQ809337.1
10	*Aspergillus ochraceus*	97	AB002068.1
11	*Cucurbitaria* sp.	99	FJ215704.1
12	*Phoma macrostoma*	97	AB454217.1
13	*Phoma* sp.	97	AY293772.1
14	*Leptosphaeria microscopica*	99	U04235.1
15	*Gondwanamyces* *proteae*	97	AY271804.1
16	*Penicillium* sp.	97	JF950269.1
17	*Monodictys arctica*	97	EU686519.1
*18*	*Graphium putredinis*	99	AB007683.1
19	*Saccobolus dilutellus*	99	FJ176814.1
20	*Orbilia auricolor*	99	DQ471001.1
21	Uncultured *soil fungi*	97	AJ877196
